# Author Correction: Tunable two-dimensional interfacial coupling in molecular heterostructures

**DOI:** 10.1038/s41467-017-01767-y

**Published:** 2017-11-17

**Authors:** Beibei Xu, Himanshu Chakraborty, Vivek K. Yadav, Zhuolei Zhang, Michael L. Klein, Shenqiang Ren

**Affiliations:** 1 Department of Mechanical Engineering, Temple University, Philadelphia, PA 19122 USA; 2 Temple Materials Institute, Temple University, Philadelphia, PA 19122 USA; 3 Department of Chemistry and Institute for Computational Molecular Science, Temple University, Philadelphia, PA 19122 USA; 4 Center for the Computational Design of Functional Layered Materials, Temple University, Philadelphia, PA 19122 USA

*Nature Communications* **8**:312 doi:10.1038/s41467-017-00390-1; Article pubilshed online: 22 Aug 2017

We regretfully omitted to give credit to a previous figure upon which the surface-tension scheme in Fig. 1b is based. The caption to Fig. 1 should have included the following: “The surface-tension scheme in Fig. 1b is adapted from Fig. 1a in Noh, J., Jeong, S. & Lee, J.-Y. Ultrafast formation of air-processable and high-quality polymer films on an aqueous substrate. *Nat. Commun.* **7**, 12374 (2016).”

