# Supplementary material for: Tunable two-dimensional interfacial coupling in molecular heterostructures
Source: Nat Commun. 2017 Aug 22;8:312. doi: 10.1038/s41467-017-00390-1 (PMC5567094; doi:10.1038/s41467-017-00390-1)
Supplement: Supplementary file 2 — Supplementary Information [file 41467_2017_390_MOESM2_ESM.pdf]

File Name: Supplementary Informations
Descriptions: Supplementary Figures, Supplementary Methods and Supplementary References

File Name: Peer Review File
Descriptions:

Supplementary Figures

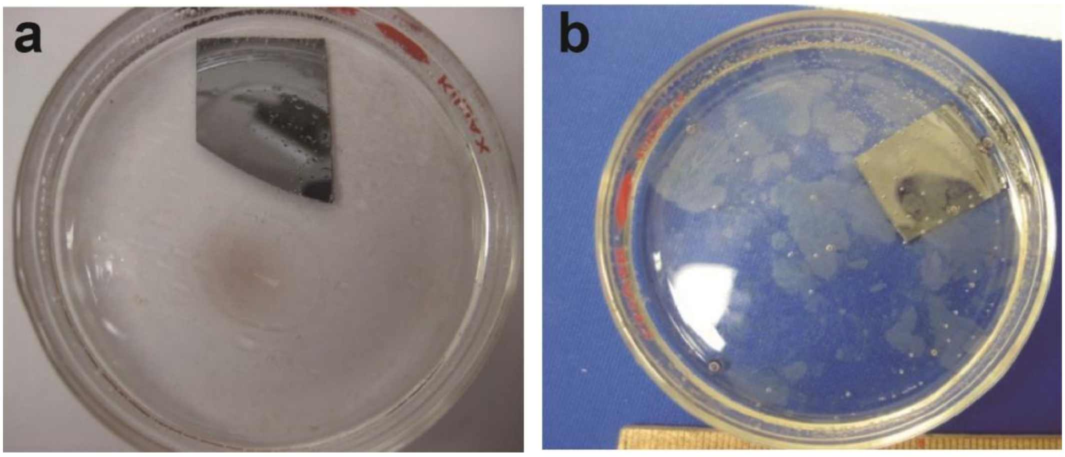

Supplementary Figure 1 Photo image of ETC_{60} nanosheet. (a) The solvent is water.

(b) The solvent is 50 v%DMF/50 v%water.

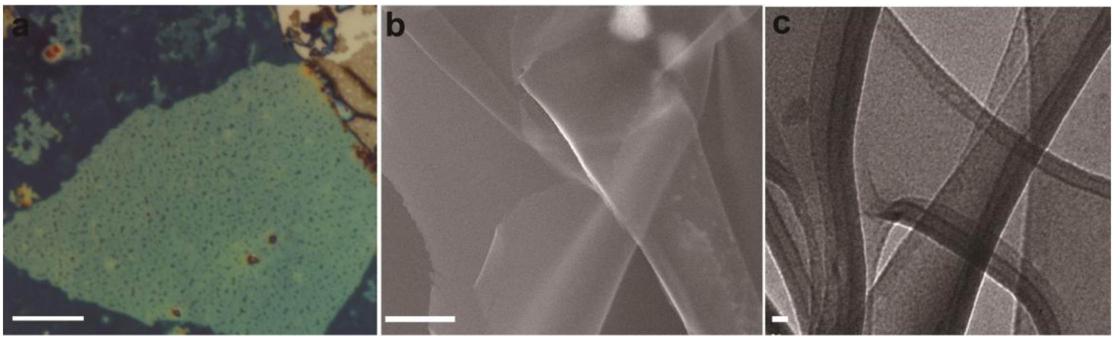

Supplementary Figure 2 Morphology of the heterostructures. (a) Optical microscopy (OM) image on Si substrate. The bottome blue layer is ETC_{60} nanosheet, the top yellow layer is DTC_{60} nanosheet. The scale bar is 100 μm. Scanning electron microscopy (SEM) image (b), and transmission electron microscopy (TEM), (c) image of the heterostructure with ETC_{60} bottome layer and DTC_{60} top layer nanosheets. The scale bar is 5 μm and 20 nm, respectively.

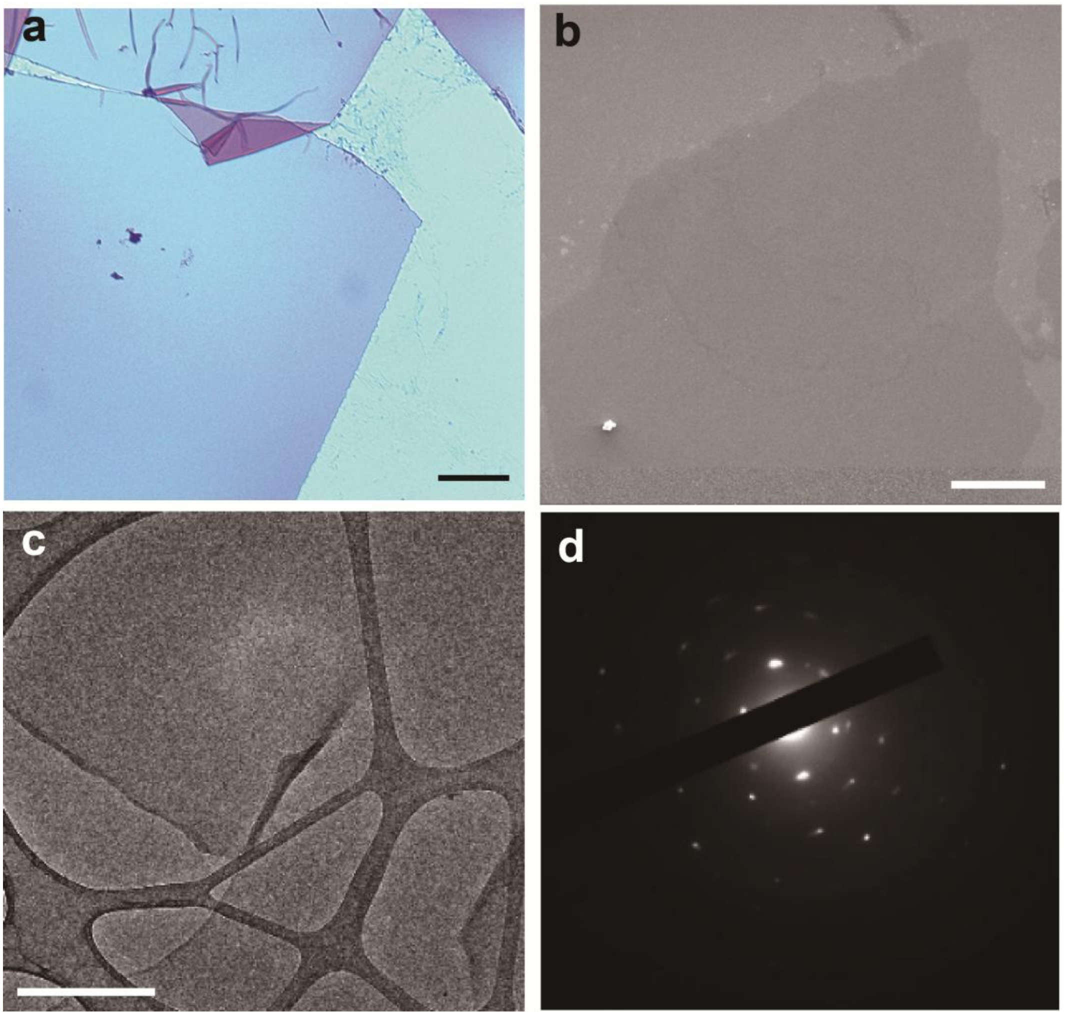

Supplementary Figure 3 Morphology of DTC_{60} nanosheet. (a-b) OM and SEM images. The scale bar is 100 μm. (c-d) TEM images and SAED pattern

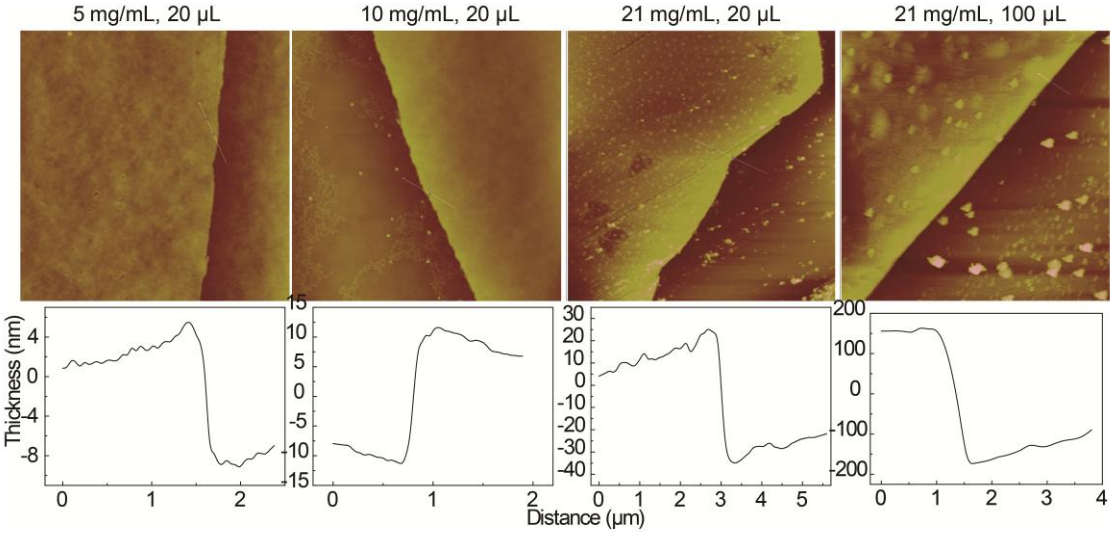

Supplementary Figure 4 AFM images of DTC_{60} nanosheet. Concentration and volume dependent thickness change.

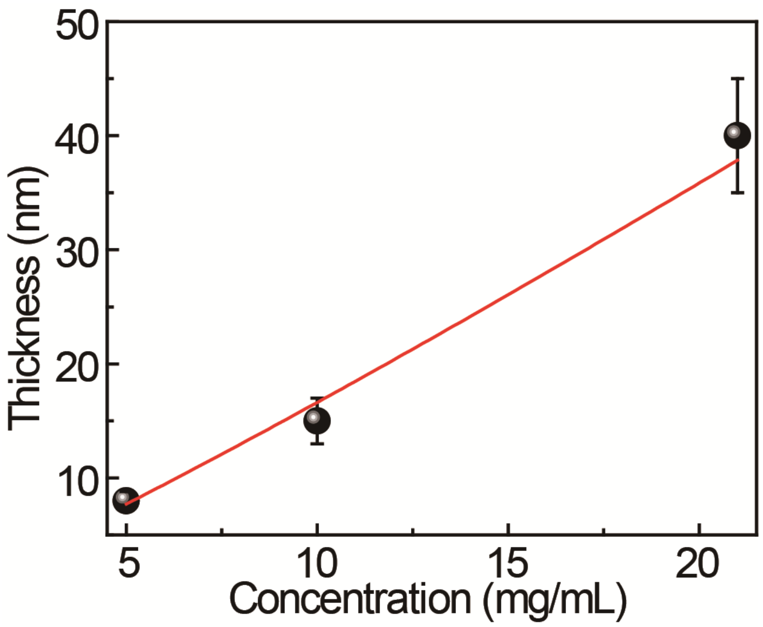

Supplementary Figure 5 Concentration dependent thickness change of DTC_{60}. By controlling the concentration and volume of organic solution, the thickness of the film can be controlled. The thickness increases from 8 to 40 nm with the increase of the concentration of DTC_{60} from 5 to 21 mg/mL at a volume of 20 μL. The further increase of the volume to 100 μL at the concentration of 21 mg/mL leads to the increase of the thickness to ~200 nm.

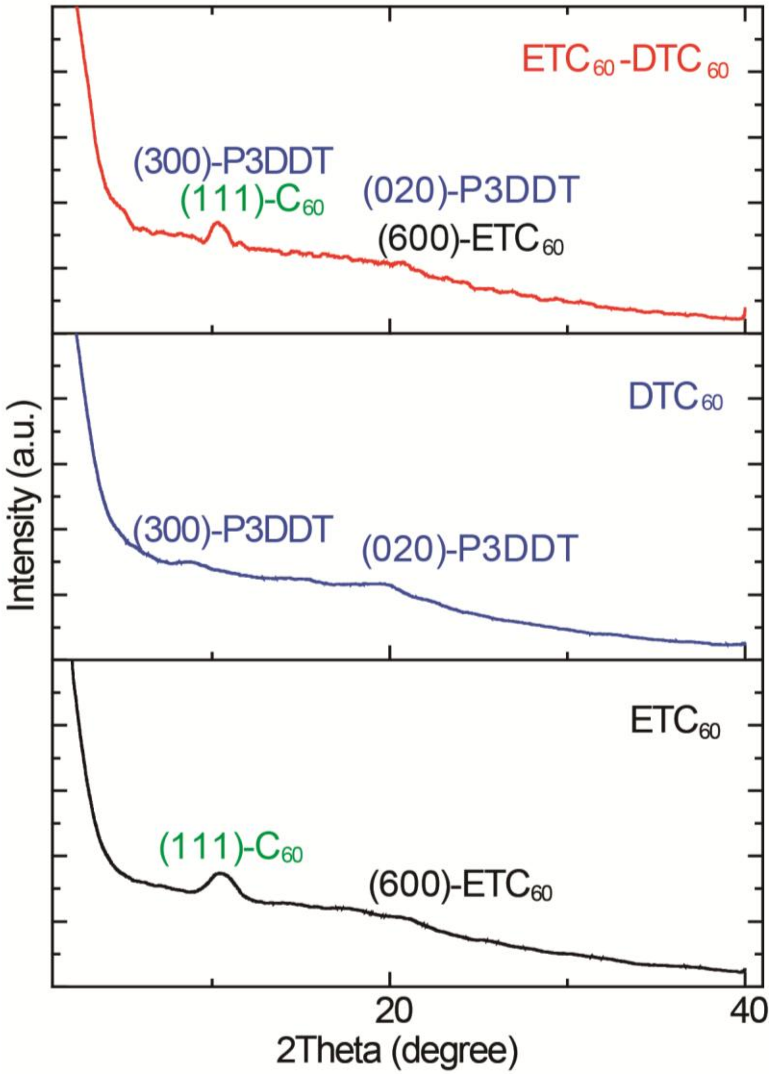

Supplementary Figure 6 XRD patterns of the nanosheets. The XRD patterns demonstrate that P3DDT (300) crystalline face of DTC_{60} are along the horizontal direction with P3DDT molecular chain in the in-plane orientation. A peak at ~21° from ETC_{60} crystal appears, demonstrating that molecular chain of BEDT-TTF is in the in-plane orientation which is consistent with the structure we mentioned in the main text. In addition, the horizontal stacking of the molecular chains is the most stable free-standing structure on the surface of water due to the side-chain interaction on the surface of water.

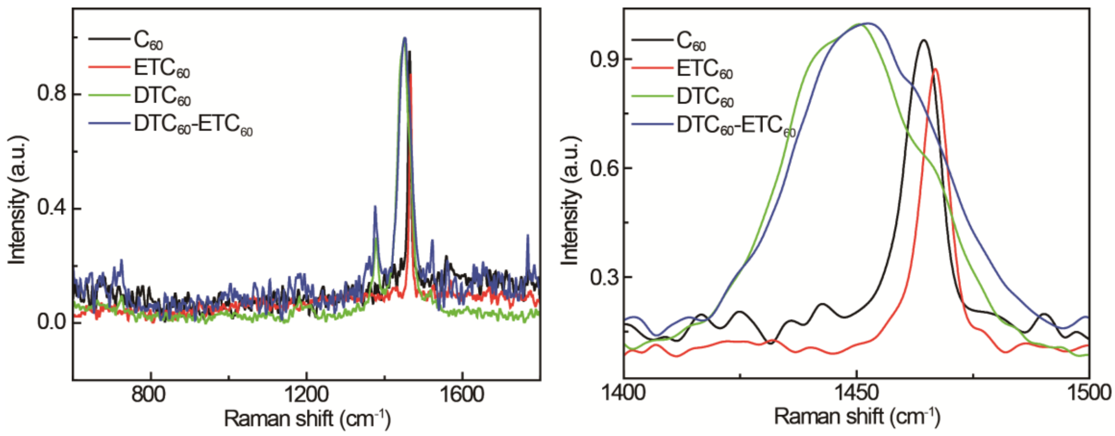

Supplementary Figure 7 Raman spectra of nanosheets. The wavelength of the laser is 532 nm laser. Symmetric (A_g) mode of C_{60} at 1465 cm^{-1} is used to test the ionicity of the charge transfer complex. The shift of A_g peak and the broadening of the peak for charge transfer complex compared to single C_{60} nanosheet confirm the formation of charge transfer crystals. The shift of A_g peak of ETC_{60}-DTC_{60} heterostructure nanosheets compared to single ETC_{60} and DTC_{60} nanosheets confirm the existence of coupling between ETC_{60} and DTC_{60} nanosheets.

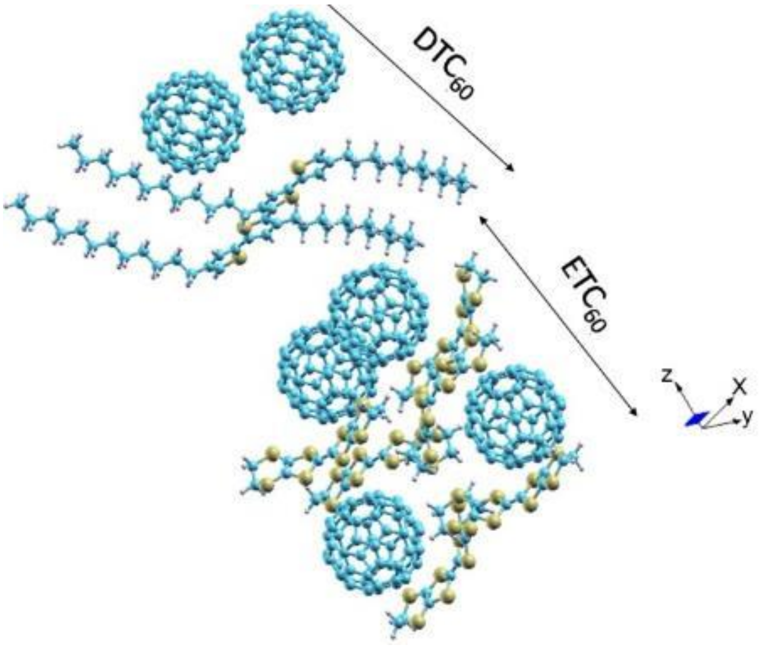

Supplementary Figure 8 Stacking structure of the heterostructure. z axis is the out-of-plane orientation. x and y axis is along the in-plane orientation.

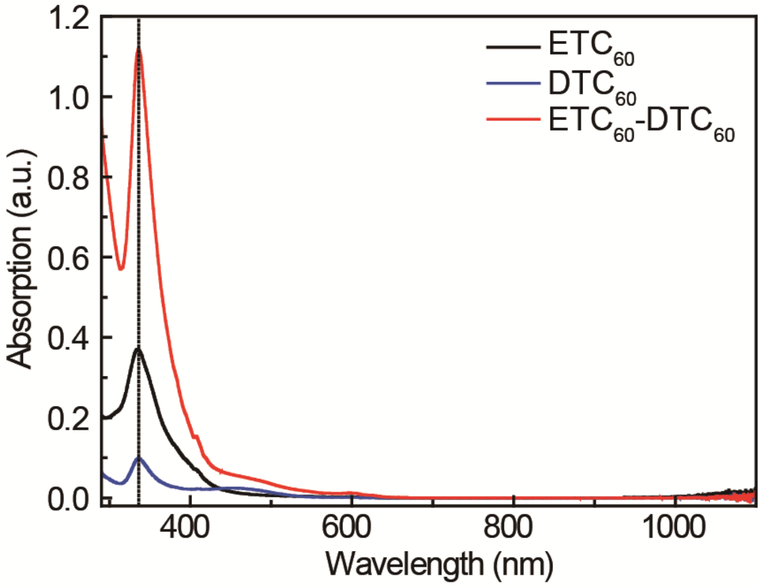

Supplementary Figure 9 Absorption spectra. The absorption spectra is obtained from BEDT-TTF/C_{60} (ETC_{60}), P3DDT/C_{60} (DTC_{60}) and their mixure (ETC_{60}-DTC_{60}). in 1,2-DCB solvent

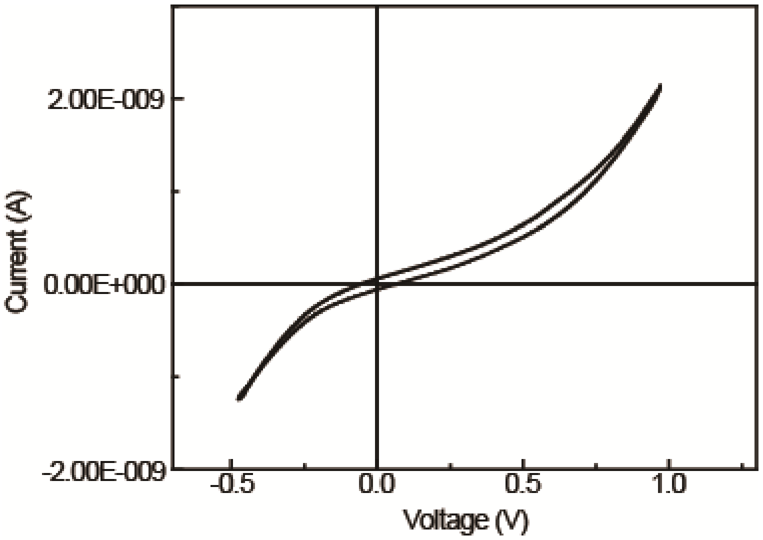

Supplementary Figure 10 Hysteresis current-voltage curve of the heterostructure. Only limited hysteresis originating from the intrinsic properties of the nanosheets can be observed, eliminating the possibility of forming charge traps by DMF solvent. As discussed above, the contact between the nanosheets and DMF/water mixed solution is monolayer. Moreover, all the organic donor and acceptor can't be dissolved in DMF. The two-dimensional compression during the formation of film can also expel DMF into water.[1] The synchrotron data also confirms that the solvent is removed from the lattice.[2]

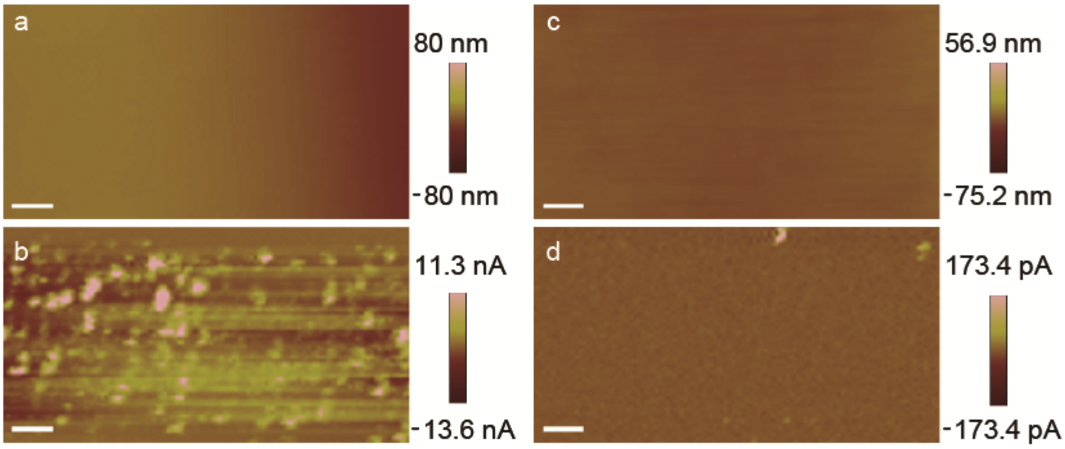

Supplementary Figure 11 Conducting-AFM images. All the images correspond to the same device measured along the horizontal (a, b) and vertical (c, d) orientations. (a) and (c) are AFM topography images. (b) and (d) are current images. The scale bar is 1 μm. The loaded voltage is 0.5 V. To exclude the influence of the structures for horizontal and vertical directions on the optoelectronic properties, we measured the conducting-AFM on the same device of the same structure to verify the anisotropic conductivity. The conductivity for horizontal orientation is much larger than that of the vertical orientation, which is consistent with the discussion in our main text. For the measurement along the horizontal orientation, the electrode is loaded in the left, thus, the left part of Supplementary Figure 11b shows high conductivity than that of the right part. However, for the vertical orientation measurement in Supplementary Figure 11d, the electrode is at the bottom, thus, the film shows homogeneous conductivity.

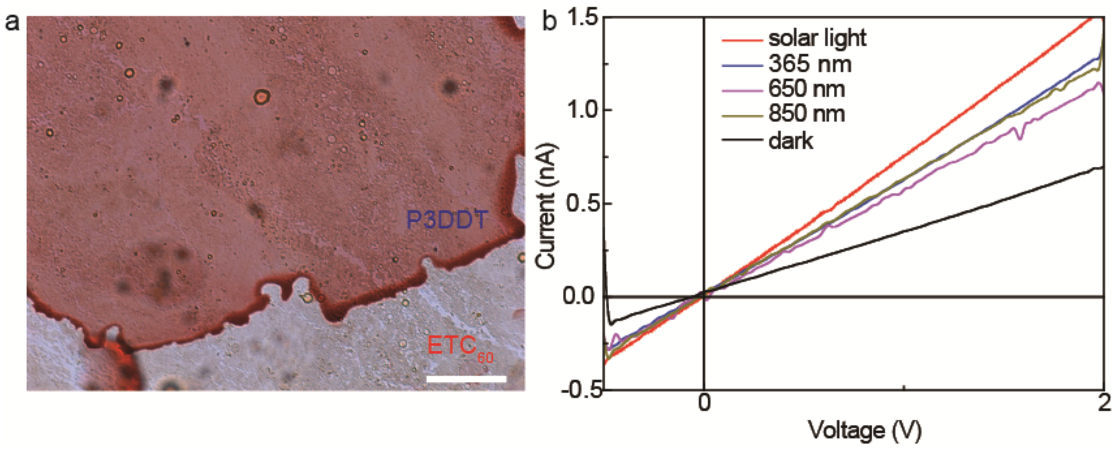

Supplementary Figure 12 Morphology and photoresponse of P3DDT/ETC$_{60}$. (a) Optical microscope. The scale bar is 100 μm. (b) Photoresponse of P3DDT/ETC$_{60}$ nanosheets under dark and light of different wavelength.

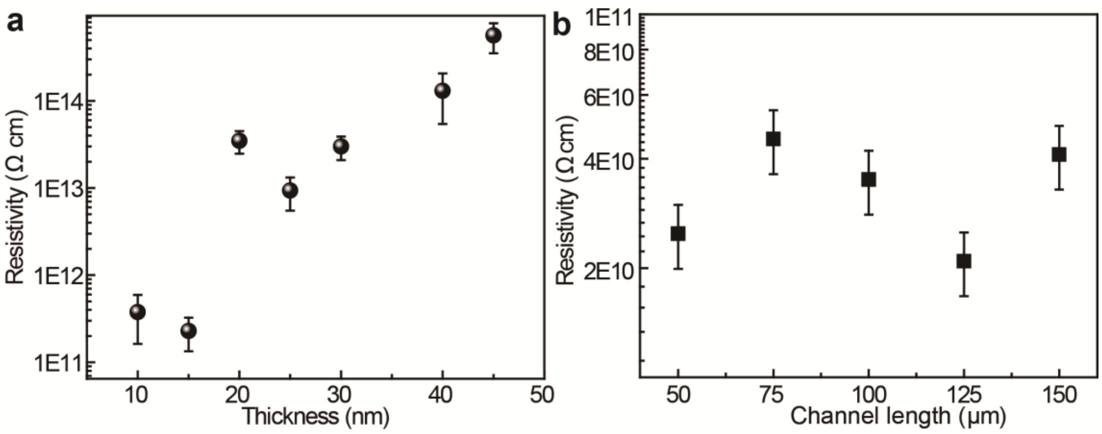

Supplementary Figure 13 Resistivity of single ETC_{60} nanosheet. (a) Thickness dependent resistivity along vertical orientation. (b) Channel length dependent resistivity along horizontal orientation. The resistivity of single layer ETC_{60} is over 100 times larger than those of ETC_{60}-DTC_{60} heterostructure, revealing the enhancement of charge transport by energy matching of the ETC_{60} and DTC_{60} energy band. Moreover, the resistivity along the horizontal orientation shows less dependence on the channel length, confirming the proposed stacking structure in Fig. 1g in the main text.

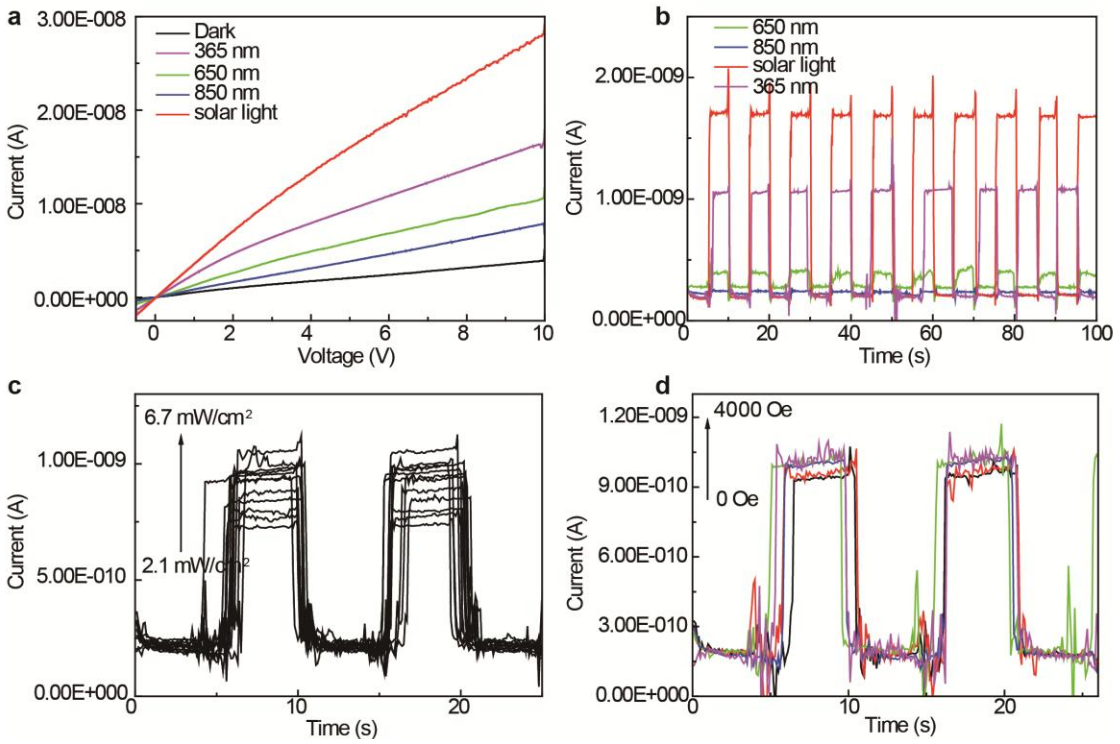

Supplementary Figure 14 Horizontal photoresonse of the heterostructure. (a) Current-voltage curves under dark and different wavelength of light illumination. (b) Photoresponse under different wavelength of light illumination. (c) Light intensity dependent photoresponse. The light wavelength is 365 nm. (d) Magnetic field dependent photoresponse under the light wavelength of 365 nm.

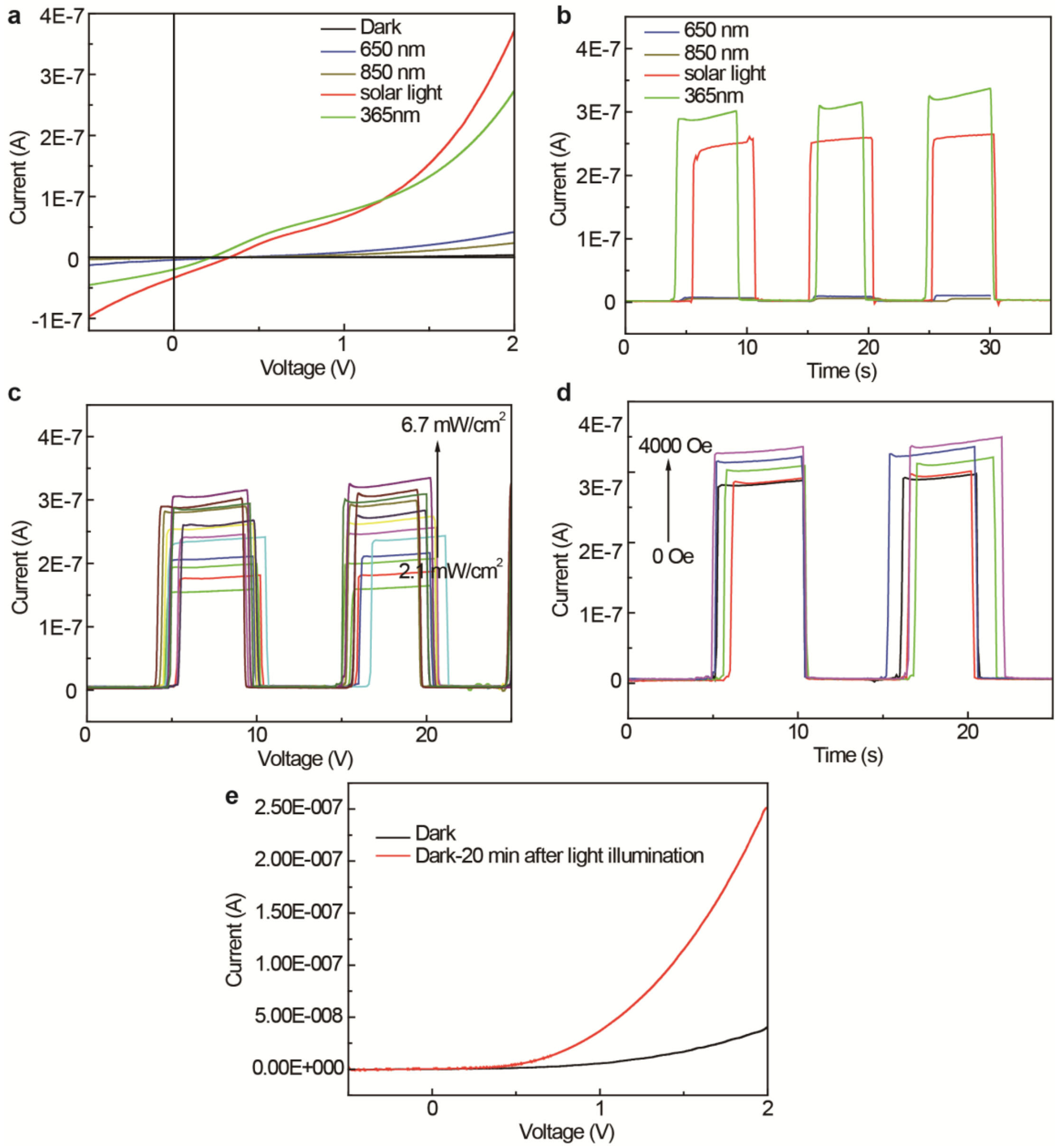

Supplementary Figure 15 Vertical photoresonse of the heterostructure. (a) Current-voltage curves under dark and different wavelength of light illumination. (b) Photoresponse under different wavelength of light illumination. (c) Light intensity dependent photoresponse. The light wavelength is 365 nm. (d) Magnetic field dependent photoresponse under the light wavelength of 365 nm. (e) Light soaking effect. The current-voltage curves is measured under dark of 20 min later after light illumination and without illumination.

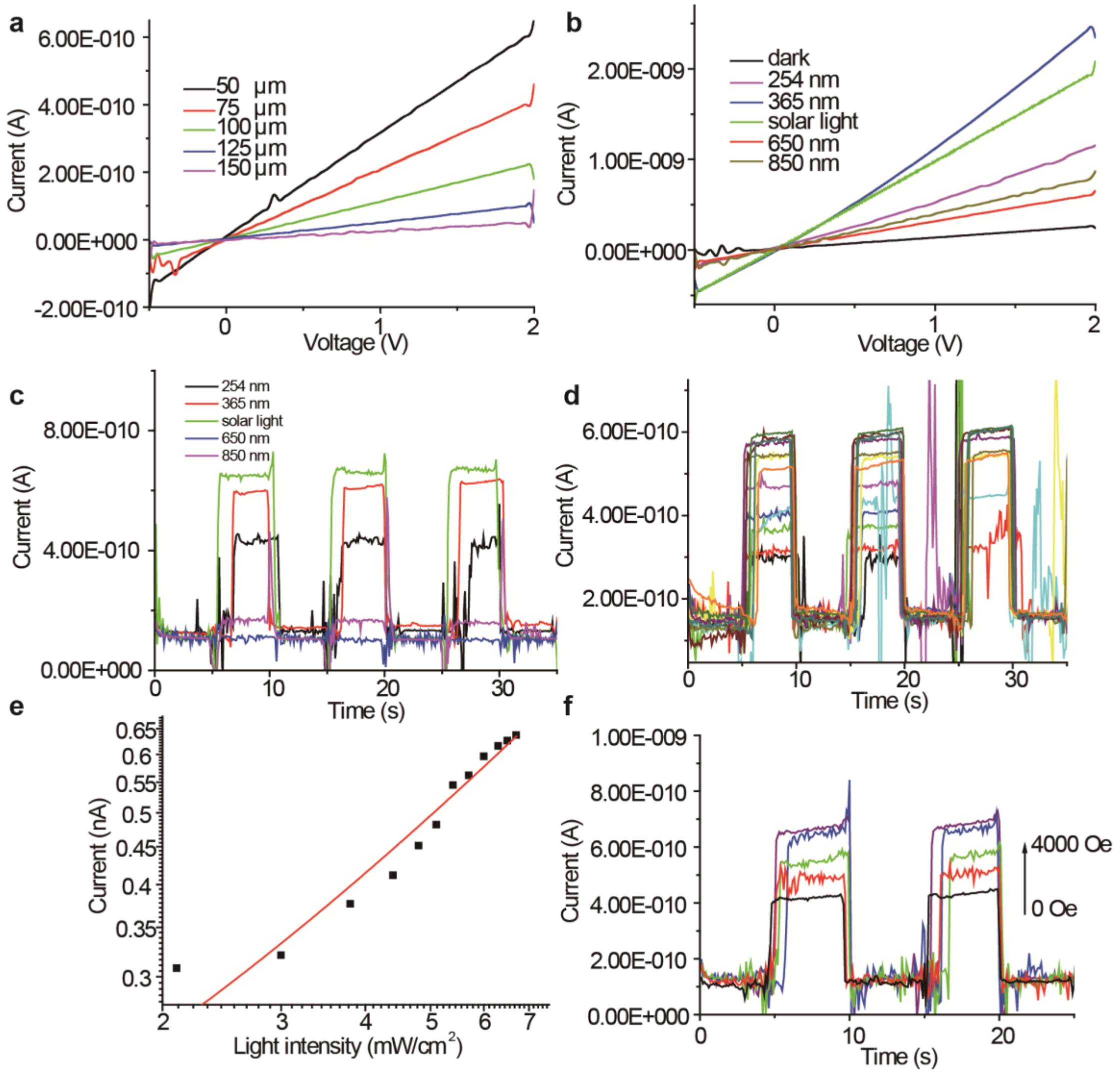

Supplementary Figure 16 Photoresonse properties of the heterostructure. The bottle layer is DTC_{60} nanosheet. The top layer is ETC_{60} nanosheet. The measurement is along the horizontal orientation. (a) Current-voltage curves with different distance channel length. The channel length are 50, 75, 100, 125 and 150 μm, respectively. (b) Current-voltage curves under dark and different wavelength of light illumination. (c) Photoresponse under different wavelength of light illumination. (d), (e) Light intensity dependent photoresponse. The light wavelength is 365 nm. (f) Magnetic field dependent photoresponse under the light wavelength of 365 nm.

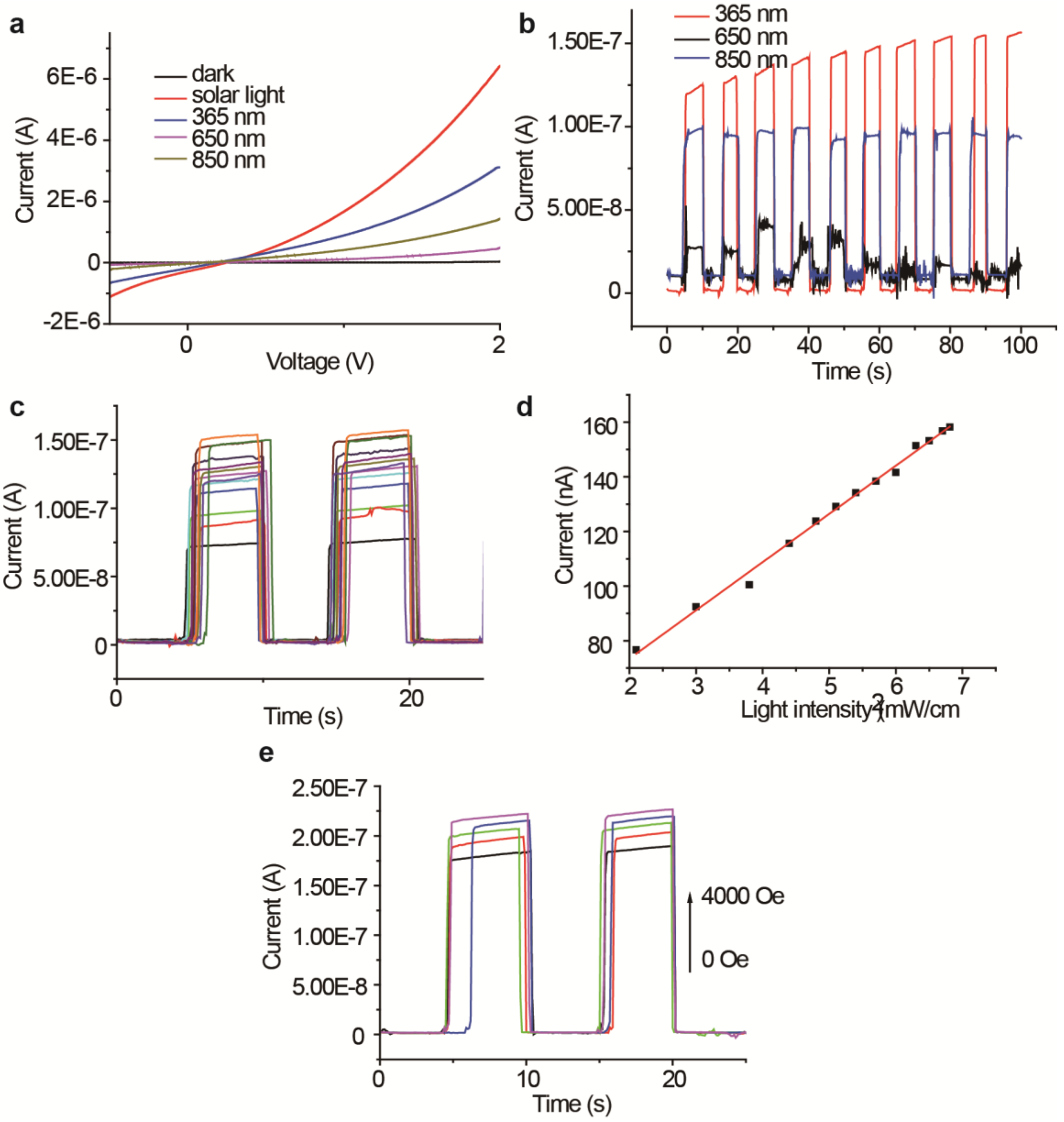

Supplementary Figure 17 Photoresonse properties of the heterostructure. The bottle layer is DTC_{60} nanosheet. The top layer is ETC_{60} nanosheet. The measurement is along the vertical orientation. (a) Current-voltage curves under dark and different wavelength of light illumination. (b) Photoresponse under different wavelength of light illumination. (c), (d) Light intensity dependent photoresponse. The light wavelength is 365 nm. (e) Magnetic field dependent photoresponse under the light wavelength of 365 nm.

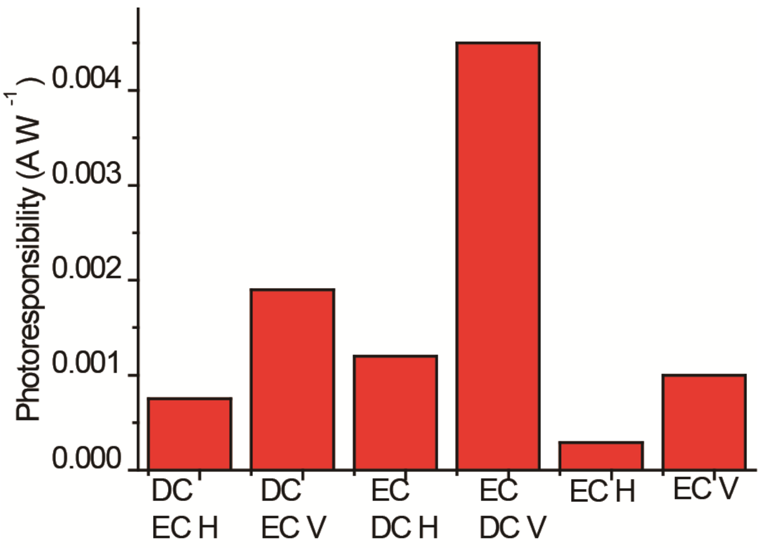

Supplementary Figure 18 Photoresponsibility of different nanosheets. The names correspond to DTC_{60} as bottom layer and ETC_{60} as top layer measured along horizontal orientation (DC EC H), DTC_{60} as bottom layer and ETC_{60} as top layer measured along vertical orientation (DC EC V), DTC_{60} as top layer and ETC_{60} as bottom layer measured along horizontal orientation (EC DC H), DTC_{60} as top layer and ETC_{60} as bottom layer measured along vertical orientation (EC DC V), ETC_{60} single nanosheet measured along horizontal orientation (EC H), ETC_{60} single nanosheet measured along vertical orientation (EC V).

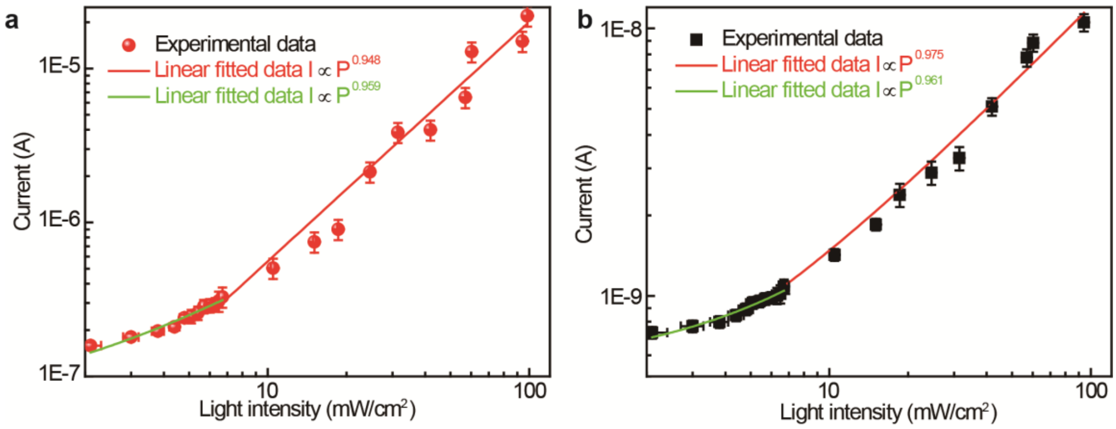

Supplementary Figure 19 Photoresponse current as a function of light intensity. (a) Vertical orientations. (b) Horizontal orientations. The photocurrent is measured up to the light intensity of 100 mW/cm^2 for both vertical and horizontal orientations. The change of linear behavior happens at ~7 mW/cm^2. As discussed in Fig. 2f of the main text, the light intensity dependent photoresponse current includes two parts, one part is below the light intensity of ~7 mW/cm^2, another part is higher than the light intensity of ~7 mW/cm^2. The two parts for the power law dependent photoresponse current follow linearly behavior $I \propto P^{\theta}$ with θ close to 1, indicating that there is few trapped photogenerated charge carriers and the recombination of charge carriers is dominated by monomolecular recombination at low light intensity.[3,4]

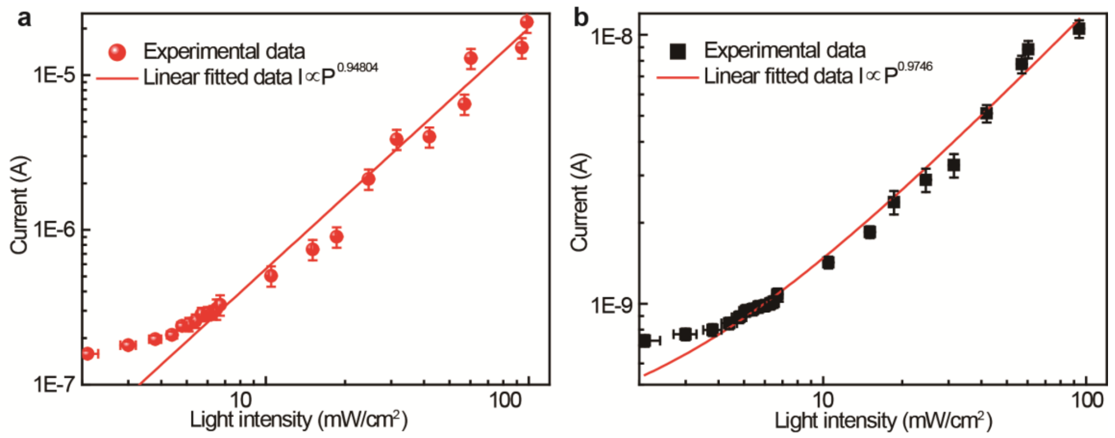

Supplementary Figure 20 Photoresponse current as a function of light intensity. (a) Vertical orientations. (b) Horizontal orientations. When the light intensity dependent photocurrent from 0 to 100 mW/cm^2 is fitted as one part, the relationship is not linear due to the loss of charge carriers via bimolecular recombination and space charge limited photocurrent from the unbalanced transport of electrons and holes.[5,6]

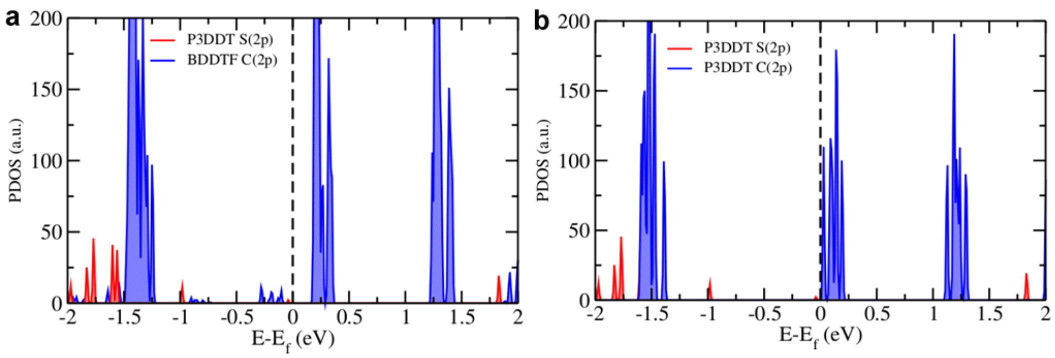

Supplementary Figure 21 Projected density of states (PDOS) plots. In Supplementary Figure 21a, the solid curves denotes the PDOS of ETC_{60}/DTC_{60} heterostructure for S (red), and dotted curve for C (blue) of the DTC_{60} complex respectively. In Supplementary Figure 21b, the solid curves denotes the PDOS of ETC_{60}/DTC_{60} heterostructure for C (red) , and dotted curve for C (blue) of DTC_{60} and ETC_{60} complex respectively. The energies have been shifted with respect to their Fermi energies.

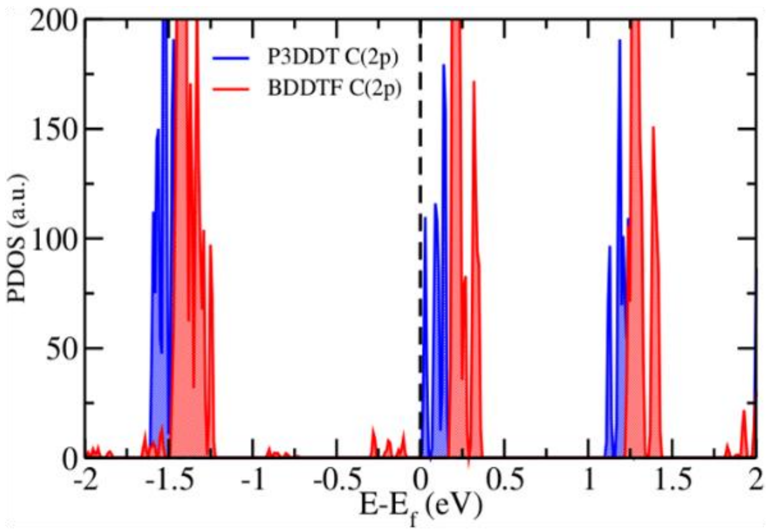

Supplementary Figure 22 Projected density of states (PDOS) plots. Solid curves denotes the PDOS of ETC_{60}/DTC_{60} heterostructure for S (red) and dotted curve for C (blue) of the DTC_{60} and ETC_{60} complex respectively. The energies have been shifted with respect to their Fermi energies.

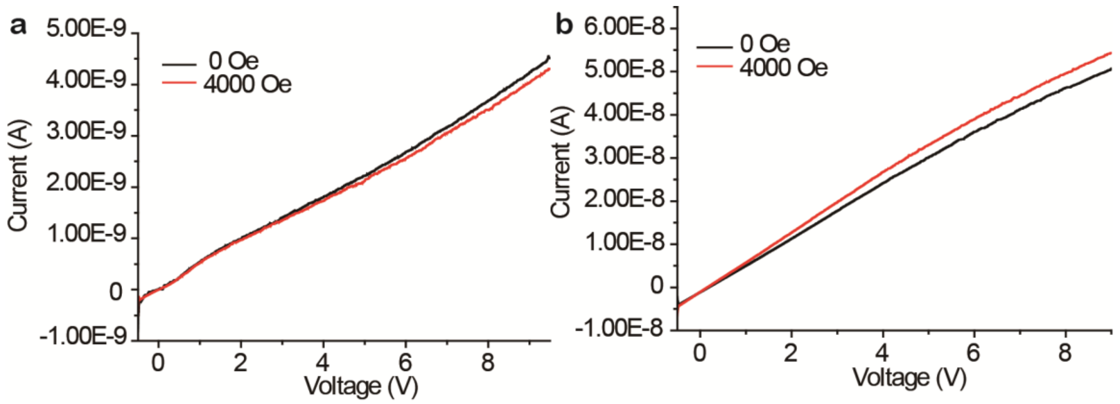

Supplementary Figure 23 Current-voltage curves with and without magnetic field. The measurement for the heterostructure is along the horizontal orientation. (a) Dark condition. (b) 365 nm light illumination.

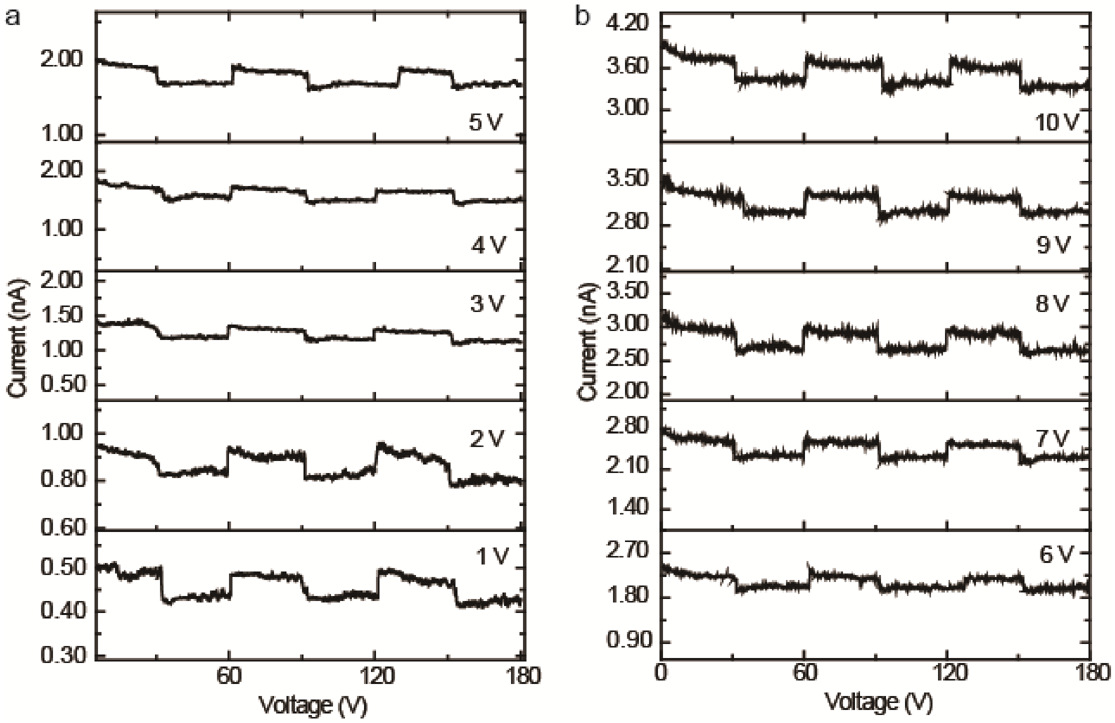

Supplementary Figure 24 Magnetic field effect on current (MC). The measurement for the heterostructure is along horizontal orientation. The voltage ranges from 1 to 10 V. The magnetic field is 4000 Oe. Magnatic field is off for the first 30 s and on for the following 30 s for each minute.

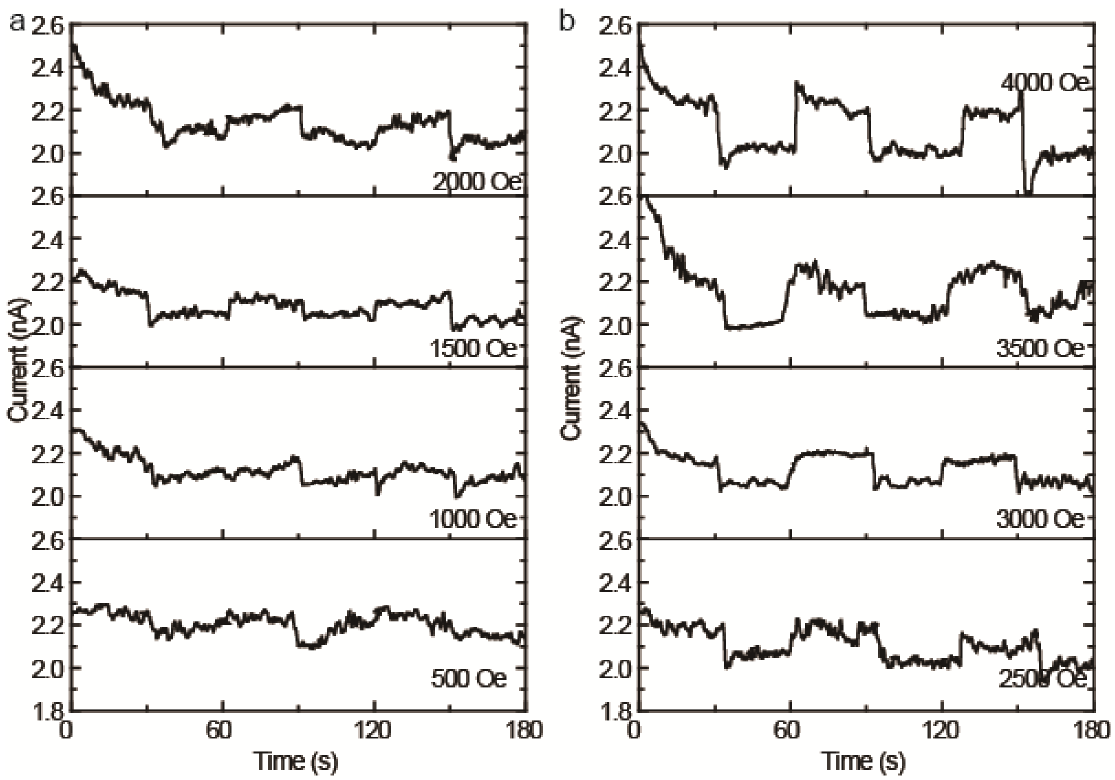

Supplementary Figure 25 MC at different magnetic field. The magnetic field changes from 500 to 4000 Oe. The measurement for the heterostructure is along horizontal orientation under 6V. Magnatic field is off for the first 30 s and on for the following 30 s for each minute.

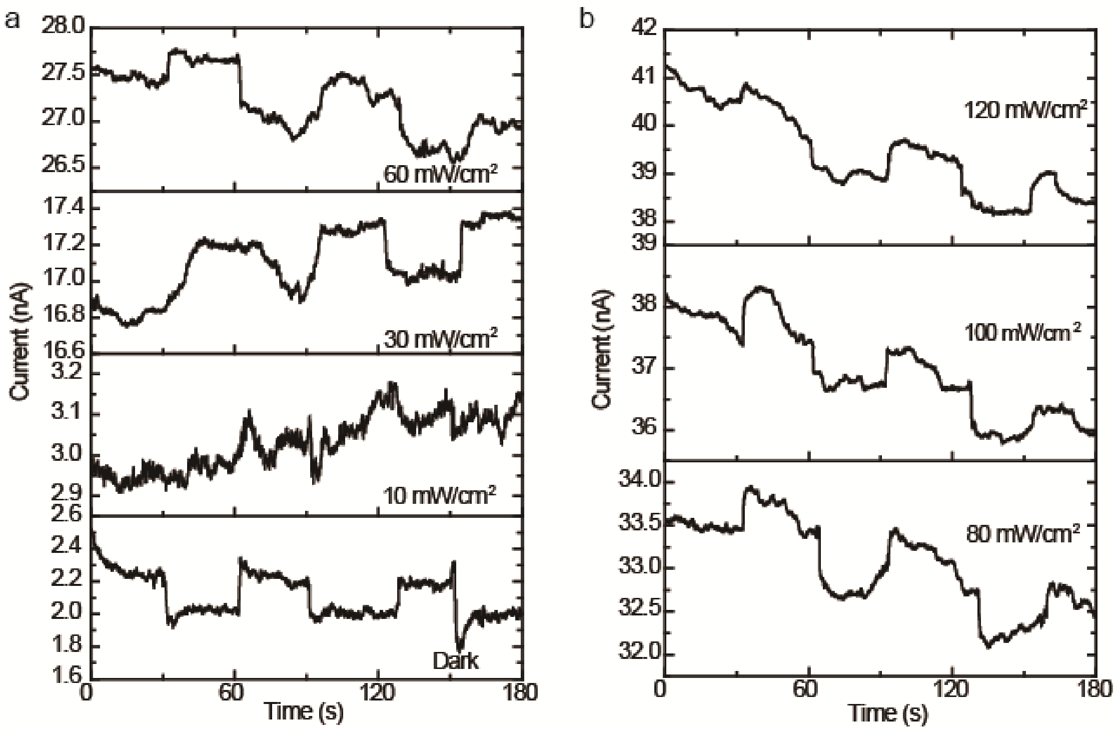

Supplementary Figure 26 MC udner different light intensity. The measurement for the heterostructure is along horizontal orientation under 6V. The light intensity ranges from 0 to 120 mW/cm^2. The magnetic field is 4000 Oe. Magnatic field is off for the first 30 s and on for the following 30 s for each minute.

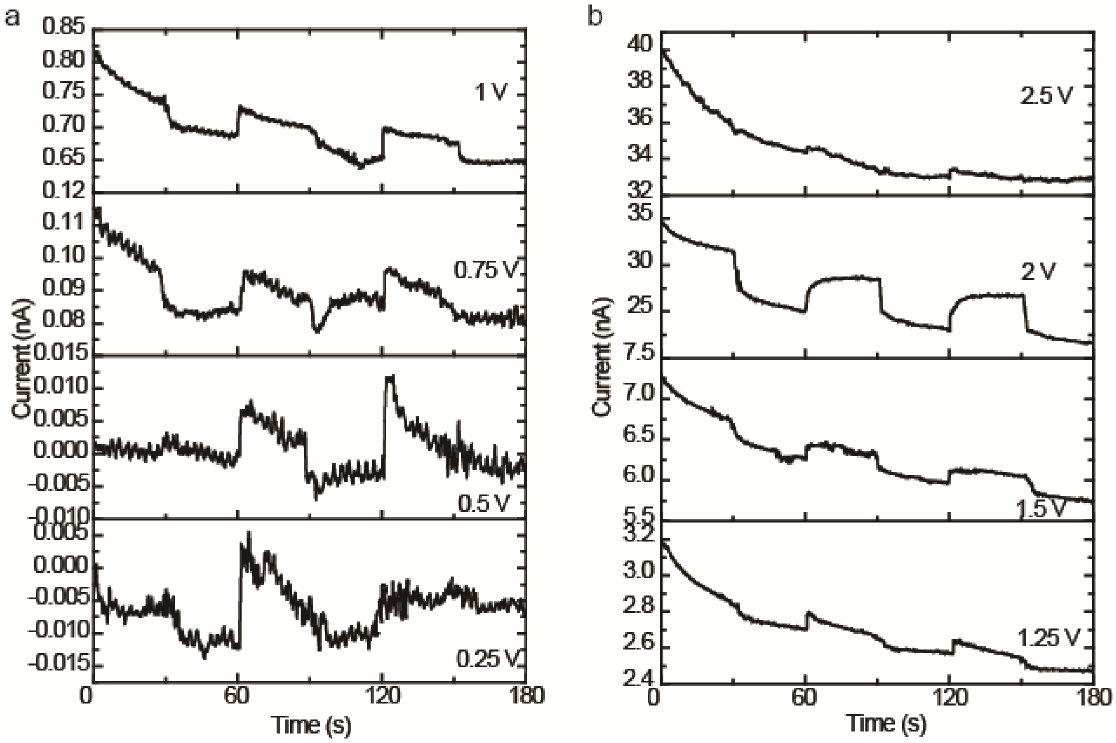

Supplementary Figure 27 MC udner different voltage. The measurement for the heterostructure is along vertical orientation. The voltage ranges from 0.25 to 2.5 V. The magnetic field is 4000 Oe. Magnatic field is off for the first 30 s and on for the following 30 s for each minute.

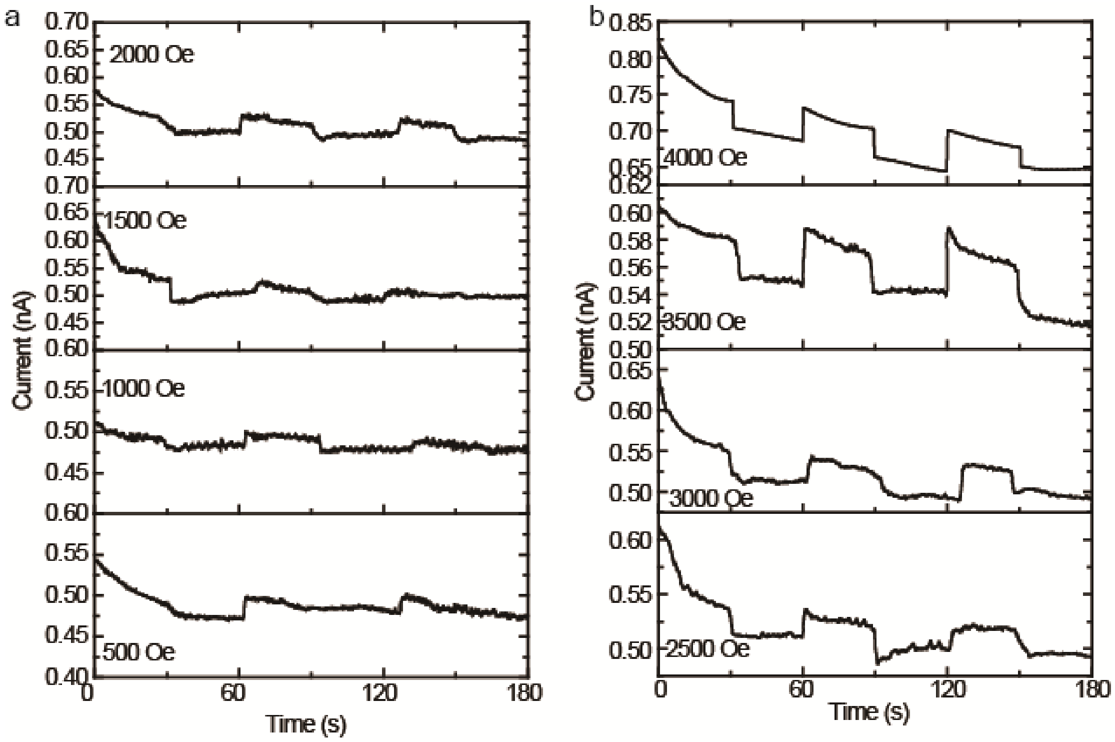

Supplementary Figure 28 MC udner different magnetic field. The measurement for the heterostructure is along vertical orientation. The voltage is 0.2 V. The magnetic field ranges from 500 to 4000 Oe. Magnatic field is off for the first 30 s and on for the following 30 s for each minute.

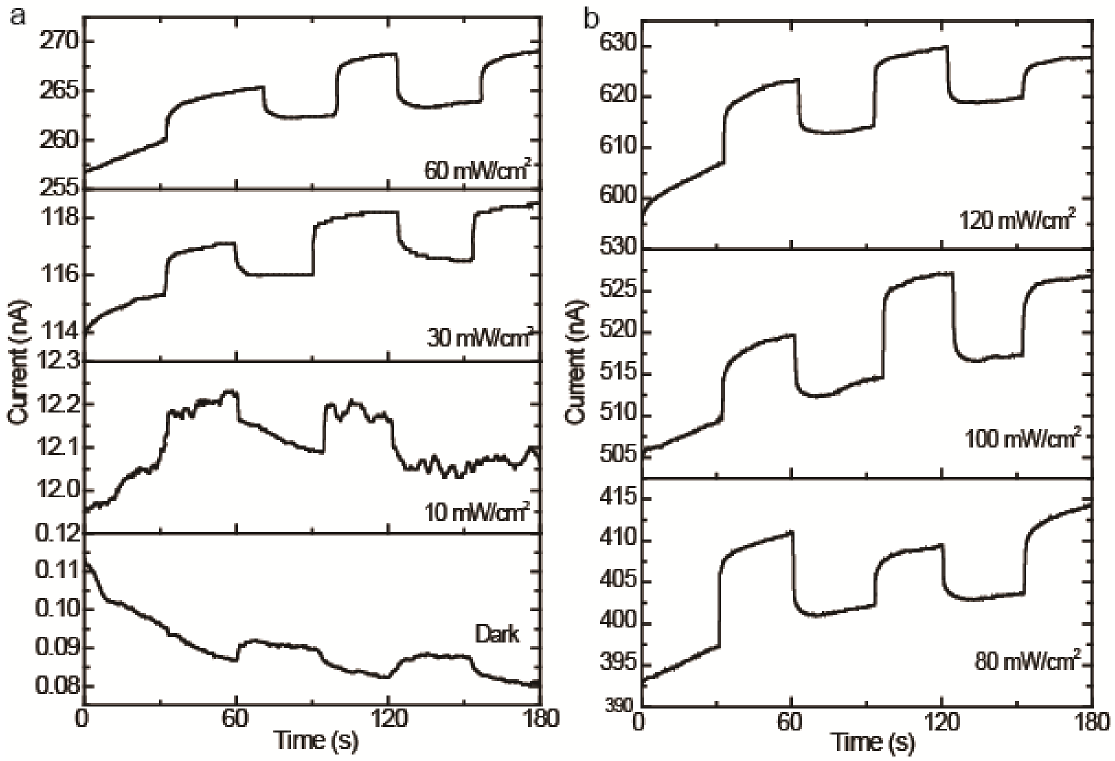

Supplementary Figure 29 MC udner different light intensity. The measurement for the heterostructure is along vertical orientation. The light intensity ranges from 0 to 120 mW/cm^2. The voltage is 0.2 V. The magnetic field is 4000 Oe. Magnatic field is off for the first 30 s and on for the following 30 s for each minute.

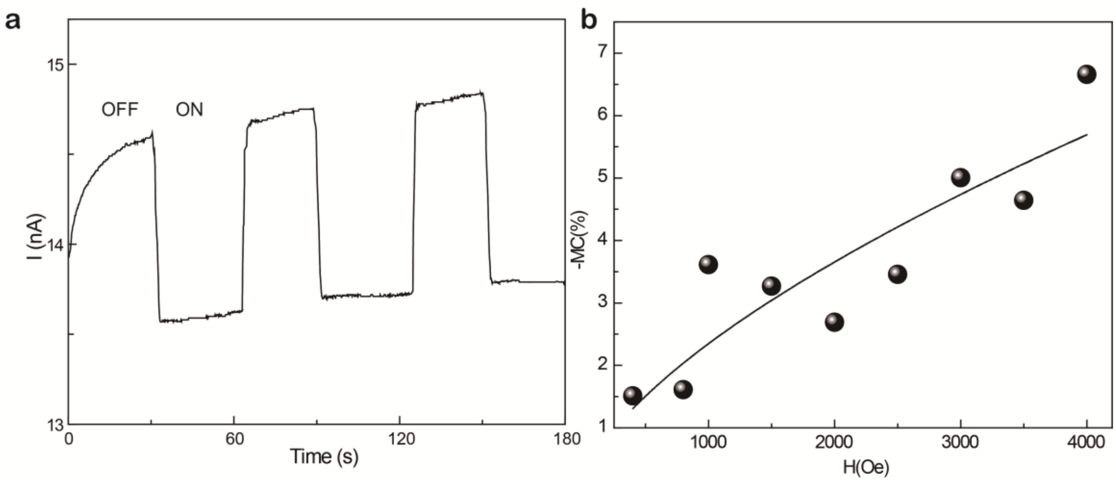

Supplementary Figure 30 MC of DTC_{60} nanosheet. (a) Current change with magnetic field ON and OFF. (b) Magnetic field dependent MC change. The DTC_{60} nanosheets have negative MC under dark condition, in which the current decreases with the loading of external magnetic field.

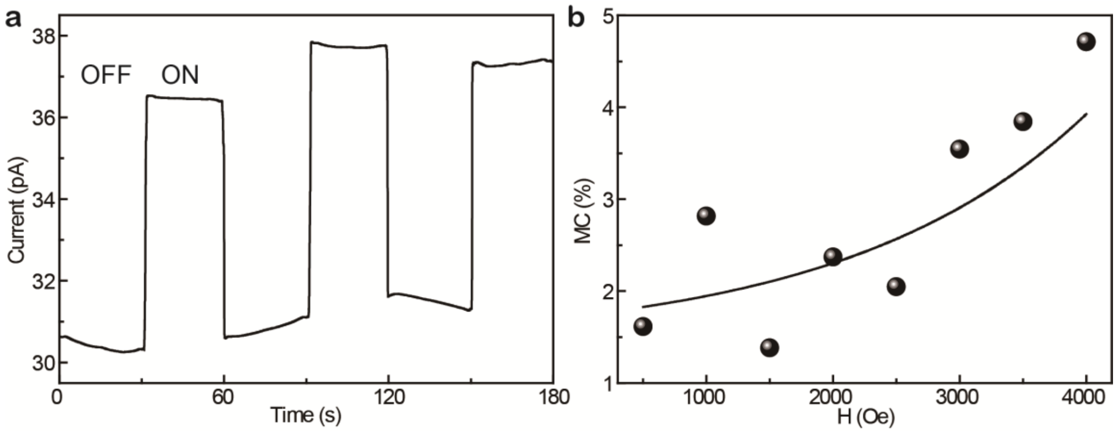

Supplementary Figure 31 MC of ETC$_{60}$ nanosheet. (a) Current change with magnetic field ON and OFF. (b) Magnetic field dependent MC change. In contrary to the MC of DTC$_{60}$ nanosheet, the ETC$_{60}$ nanosheet has positive MC under dark. As discussed in Fig. 4 of the main text, for ETC$_{60}$-DTC$_{60}$ heterostructure, it only shows negative MC under dark, thus the interaction of C$_{60}$ layer from ETC$_{60}$ nanosheet and DTC$_{60}$ nanosheet and the P3DDT layer from DTC$_{60}$ nanosheet have larger influence on the MC performace of the whole device.

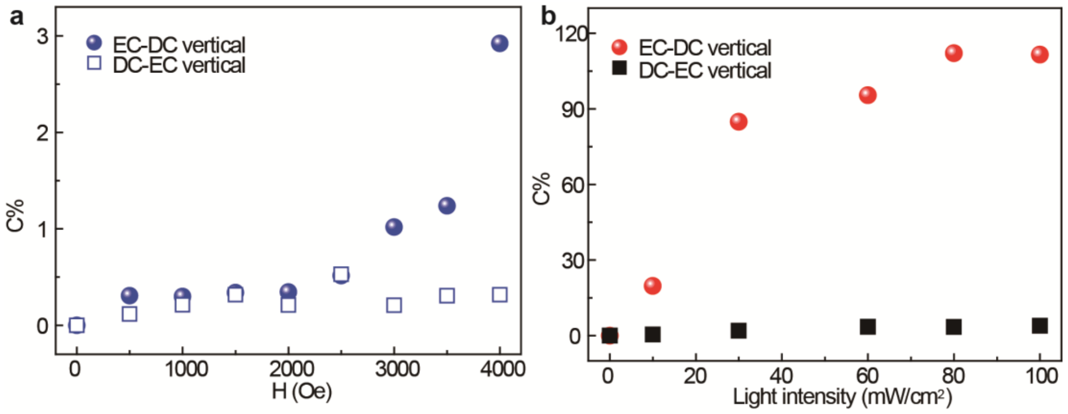

Supplementary Figure 32 Capacitance change of the heterostructures. The measurement is along vertical orientation with DTC_{60} as bottom layer and ETC_{60} as top layer (DC-EC) or ETC_{60} as bottom layer and DTC_{60} as top layer (EC-DC), respectively. (a) Magnetic field dependent capacitance. (b) Light intensity dependent capacitance.

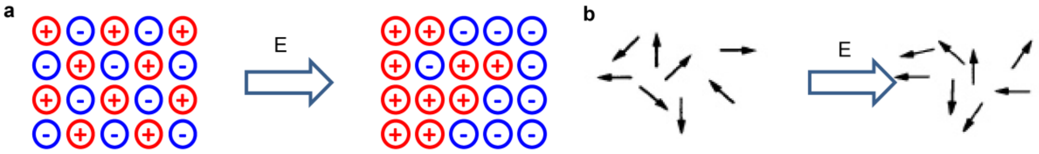

Supplementary Figure 33 Schematic figure for the mechanism of polarization. (a) Space charge polarization. (b) Dipolar polarization. With the increase of frequency, dielectric properties go through four polarization processes: 1~10^4 Hz, interfacial and space charge polarization; 10^4~10^{10} Hz, dipolar polarization; 10^{10}~10^{14} Hz, ionic polarization; 10^{14}~10^{18} Hz, electronic polarization. As our measurement is between 1~10^7 Hz, we will discuss the first two polarization processes. At low frequency, a number of conduction mechanisms (different species of charge carriers and different carrier mobilities) for the charges to accumulate at interfaces correspond with charges moving towards opposite direction, forming interfacial or space charge polarization (Supplementary Figure 33a). At high frequency, the charge cannot follow the switch of ac electric field. Only dipolar can been polarized. However, as shown in Supplementary Figure 33b, there is only a limited rotation of dipolar side groups leading to low dielectric constant.

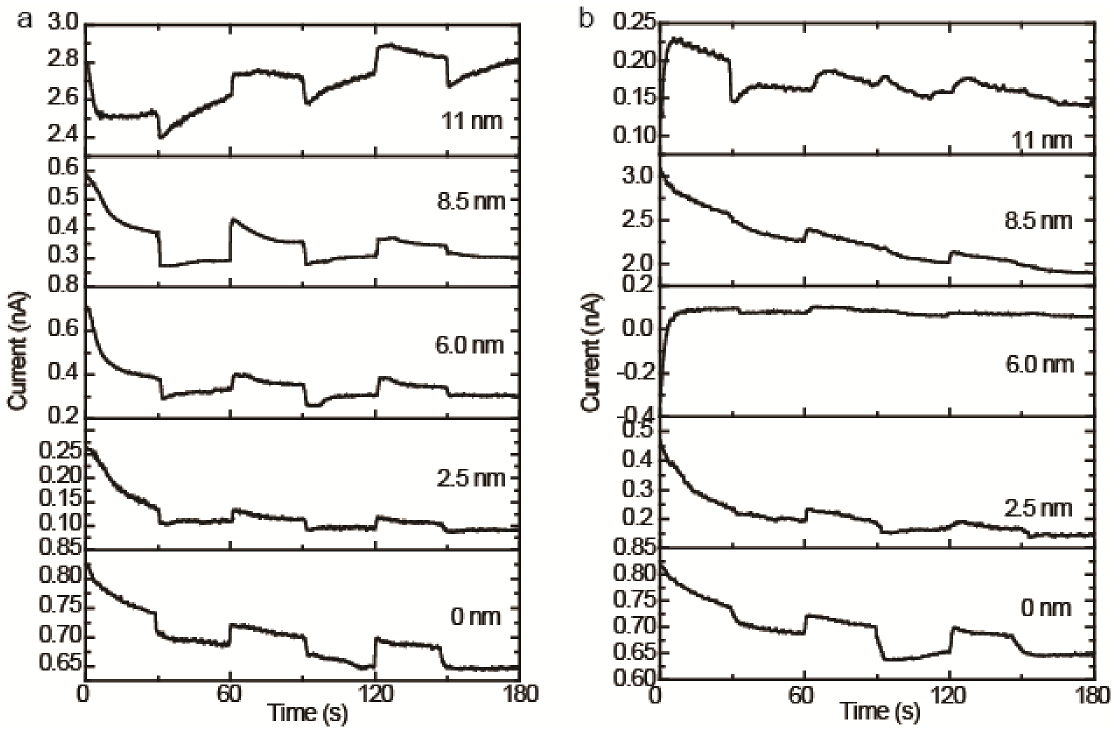

Supplementary Figure 34 MC effect for different thickness of P(VDF-TrFE). (a) P(VDF-TrFE) is underneath ETC_{60} nanosheet. (b) P(VDF-TrFE) is between ETC_{60} nanosheet and DTC_{60} nanosheet.

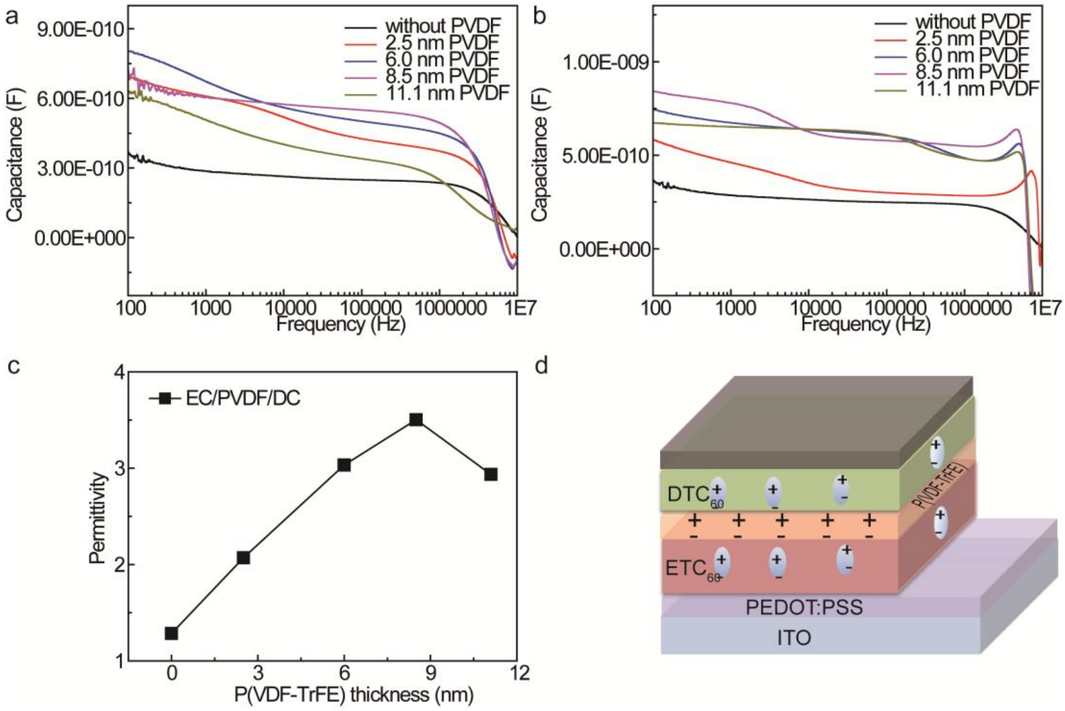

Supplementary Figure 35 The influence of capacitance by P(VDF-TrFE). The thickness of P(VDF-TrFE) ranges from 0 to 11.1 nm. (a) P(VDF-TrFE) is underneath ETC_{60} nanosheet ($PVDF/ETC_{60}/DTC_{60}$). (b) P(VDF-TrFE) is between ETC_{60} nanosheet and DTC_{60} nanosheet ($ETC_{60}/PVDF/DTC_{60}$). For $ETC_{60}/PVDF/DTC_{60}$ samples of all thicknesses (Supplementary Figure 35b), the relaxation is slower than that of the $PVDF/ETC_{60}/DTC_{60}$ (Supplementary Figure 35a) and the heterostructure without P(VDF-TrFE), due to the influence of ferroelectric field on both up DTC_{60} nanosheets and underneath ETC_{60} nanosheets. Two transformation peaks at 2000 Hz and 4.5 MHz appear for $ETC_{60}/PVDF/DTC_{60}$ sample, probably originating from the absence of coupling between DTC_{60} and ETC_{60} nanosheets due to the separation by P(VDF-TrFE). (c) P(VDF-TrFE) thickness dependent dielectric constant change of $ETC_{60}/PVDF/DTC_{60}$. It reaches highest value at the thickness of 8.5 nm. (d) Schematic illustration of the dipole alignment of $ETC_{60}/PVDF/DTC_{60}$.

Supplementary methods

Preparation of the two-dimensional molecular heterostructure devices. Patterned ITO substrates were cleaned as the method reported previously.[7] Poly(9,9-dioctylfluorene-co-N-(4-(3-methylpropyl))diphenylamine) (TFB) was used as hole transport layer and dissolved at 3 mg/mL in p-xylene solvent at 60 ^{o}C for 3 h. For the devices measured along vertical orientation, TFB was spun-coated on the patterned ITO substrates at 3400 rpm/min, and dried at 180 ^{o}C for 1h in glove box. After the formation of BEDT-TTF/C_{60} (ETC_{60}) and P3DDT/C_{60} (DTC_{60}) nanosheets according to the methods in the main text, the substrates were immersed into the mixed solvents (distilled water/dimethylformamide) beneath the ETC_{60} nanosheets at an angle of 30°, and slowly pulled out of the solvents at 2 mm/min. Then the ETC_{60} nanosheets on the substrates were annealed at 60 ^{o}C for 2 h in glove box. The same procedure was repeated to obtain DTC_{60} nanosheets. After annealing at 60 ^{o}C for 2 h again, the whole devices were annealed at 120 ^{o}C for 20 min. So, the bottom layer of the heterostructure for the device is ETC_{60} nanosheet, while the top layer is DTC_{60} nanosheet. Then, thermal evaporation was used to deposit a small molecular layer of bathocuproine (BCP) at a thickness of 7 nm and a top layer of Al metal electrode of 100 nm. For the measurements along horizontal orientation, bare substrates with patterned parallel ITO electrodes at a distance of 50 μm were used to transfer the nanosheets. Then, the nanosheets were at annealed 60 ^{o}C for 2 h in glove box.

Preparation of P(VDF-TrFE) layer. P(VDF-TrFE) was dissolved in dimethylformamide at 4 mg/mL and stirred at 60 ^{o}C for 5 h in glove box. For the

devices with P(VDF-TrFE) layer underneath ETC_{60} nanosheets, Poly(3,4-ethylenedioxythiophene)-poly(styrenesulfonate) (PEDOT:PSS) was used as the hole transport layer spun coated on patterned ITO substrate at 3600rpm for 1 min and dried at 150 ^{o}C for 0.5 h in air. For the devices with P(VDF-TrFE) layer between ETC_{60} and DTC_{60} nanosheets, TFB was used as the hole transport layer. P(VDF-TrFE) solution was spun coated at 3000, 4000, 5000 and 6000 rpm for 1 min to get different thickness of film. Then, they were annealed at 135 ^{o}C for 1 h in glove box.

Preparation of flexible devices on PDMS. PDMS pre-polymer and curing agent were mixed thoroughly at a weight ratio of 10:1 on a glass substrate at a thickness smaller than 0.5 mm. Then, they were put into a dessicator and degassed for 20 min. The mixture was annealed in an oven at 70 ^{o}C for 5 min in air, after which a copper wire with a diameter of 30 μm was pasted on the wet surface of the mixture with one end embeded in the mixture. Then, the whole mixture with copper wire was dried in the oven again for 3 h. The dried PDMS membrane with copper wire on its surface was thermally deposited with silver electrodes of 200 nm under a low heat power. Then, the PDMS membrane was peeled off from the glass substrate and pasted onto the surface of a new thick PDMS pre-polymer with curing agent mixture again. The thickness of the dried PMDS substrate is ~1.5 mm. The thick PDMS with silver electrodes was then treated by oxygen plasma for 5 min. After that, they were used as substrates for the transfer of nanosheets as discussed above.

Supplementary References

1. Guennouni, Z. et al. Self-Organization of Polystyrene-b-polyacrylic Acid (PS-b-PAA) Monolayer at the Air/Water Interface: A Process Driven by the Release of the Solvent Spreading. *Langmuir* **32**, 1971 (2016).
2. Xu, B. *et al.* Solution-Processed Molecular Opto-Ferroic Crystals. *Chem. Mater.* **28**, 2441-2448 (2016).
3. Kind, H., Han, Y., Messer, B., Yang, P., Nanowire Ultraviolet Photodetectors and Optical Switches. *Adv. Mater.* **14**, 158 (2002).
4. Koster, L. J. A., Mihailetchi, V. D., Xie, H., Blom, P. W. M. Origin of the light intensity dependence of the short-circuit current of polymer/fullerene solar cells. *Appl. Phys. Lett.* **87**, 203502 (2005).
5. Koster, L. J. A., Mihailetchi, V. D., Blom, P. W. M. Bimolecular recombination in polymer/fullerene bulk heterojunction solar cells. *Appl. Phys. Lett.* **88**, 052104 (2006).
6. Mihailetchi, V. D., Wildeman, J., Blom, P. W. M. Space-Charge Limited Photocurrent. *Phys. Rev. Lett.* **94**, 126602 (2005).
7. Xu, B., *et al.* All-polymeric control of nanoferronics. *Sci. Adv.* **1**, e1501264 (2015).
